# The web of conflict-related interactions in Colombia: exploring causal linkages between ecological and social variables by the qualitative loop analysis

**DOI:** 10.1098/rstb.2023.0165

**Published:** 2024-07-22

**Authors:** Antonio Bodini, Jenny Vivian, Juan Vargas, Nicola Clerici, Marco Scotti

**Affiliations:** ^1^ Department of Chemistry, Life Sciences and Environmental Sustainability, University of Parma, Parma 43124, Italy; ^2^ Forest Research Institute, University of Sunshine Coast, Sippy Downs, Queensland, Australia; ^3^ Department of Socio-Economic Mathematical and Statistical Sciences ESOMAS, School of Management and Economics, University of Turin, Corso Unione Sovietica, 218 bis, Turin 10134, Italy; ^4^ Department of Biology, Faculty of Natural Sciences, Universidad del Rosario, Bogotá, DC, Colombia; ^5^ GEOMAR Helmholtz Centre for Ocean Research Kiel, Kiel, Germany; ^6^ Institute of Biosciences and Bioresources, National Research Council of Italy, Sesto Fiorentino, Firenze 50019, Italy

**Keywords:** Colombia, causal linkages, complexity, conflict, loop analysis, socio-ecological systems

## Abstract

In Colombia, the long-lasting internal conflict heavily shaped the socio-ecological context and imposed relationships that persisted after the peace agreement was signed in 2016. One question of interest is whether policies or interventions conceived to attain desirable goals for the post-conflict society may be effective or, rather, if the constraints imposed by the conflict scenario might produce unintended effects, either on the environmental or the social side. To explore this issue, we envisaged the socio-ecological system as a parsimonious set of characteristic ecological and social variables within the conflict-related framework and reconstructed their interactions, exploiting elicitation-based information and the literature. We visualized the resulting interactive networks as signed digraphs. Applying the qualitative technique of loop analysis combined with numerical simulations, we predicted the response of the system to policies as drivers of change, such as subsidized credit to capital-intensive activities or policies that increase small farming competitiveness and access to markets. Highlighting causal linkages reveals that the persistence of conflict factors may produce unexpected interdependencies between licit and illicit activities and that, only in a few cases, the persistence of these mechanisms allows synergies between desirable goals.

This article is part of the theme issue ‘Connected interactions: enriching food web research by spatial and social interactions’.

## 1. Introduction

Colombia is at a crossroads. After more than 50 years of internal armed conflict, the peace agreement signed between the government and the Revolutionary Armed Forces of Colombia (FARC from the Spanish acronym) in 2016 has been welcomed to mark the beginning of a socially just and environmentally friendly developmental pathway for the country [1]. However, difficulties in enforcing the peace accord [2,3] produced adverse effects. While FARC put down their weapons, other insurgent or criminal groups have taken control over their territory. The persistence of belligerent factions [4] reiterates typical conflict-dependent dynamics, triggering impoverishment of rural populations, forced displacements, land grabbing, increased inequality, deforestation and environmental degradation [5–8].

Natural resources, forests in particular, after being arguably one of the most important sources of fuel for the conflict [9,10], have been called upon as the pillars for a more sustainable future [11]. Since the peace agreement, the rate of deforestation in Colombia has increased by 44% [12], also targeting protected areas [13]. Increased deforestation, however, cannot be classified simply as the outcome of the typical business-as-usual exploitation of the forested areas that were previously controlled by FARC. It depends on the interplay between factors linked to the conflict, such as armed groups that finance their activity through growing illicit crops at the expense of forests; victims of displacement that often settle in forested areas to create new plots to cultivate; and the existence of a grey zone in which legal business merges with illicit activities and favours deforestation and natural resource exploitation [14–20].

Forests are tied to conflict, violence and illegal, as well as legal, activities, and these ties are complex [21]. This article is an attempt to explore some of these ties and their subjacent complex dynamics. To this end, we have focused on the socio-ecological system that embeds forests, with an emphasis on the network of interactions that conflict-related factors and illegality have shaped, to unveil the possible consequences that this network may bring about. Our analysis is not focused specifically on deforestation; rather, it deals with how the structure of the interactions may modulate the response of the whole system as well as that of single components (including forests) to drivers of change, such as policies enforced or interventions conceived to attain certain management goals.

Specific objectives of this investigation include: (i) the shaping of a plausible web of the socio-ecological interactions that includes conflict-related factors; (ii) its analysis to predict the response of the variables to external drivers, such as policies or management interventions; (iii) the reconstruction, based on variables’ responses to drivers, of the potential causal linkages between conflict-related factors and socio-ecological dynamics; and (iv) the understanding of the interdependencies to highlight potential synergies and trade-offs between ecological and social goals. To achieve these objectives, we selected a parsimonious set of variables among the social and environmental components that the literature designated as crucial in the conflict dynamics. Then, we mapped out their interactions and obtained a series of networks that we analysed using loop analysis [22], a qualitative technique particularly useful in data-poor contexts [23,24].

A system-thinking analysis of the complex socio-ecological interactions in the Colombian contexts is not new [25]. Arias-Gaviria *et al*. [26] implemented a whole system approach to understand deforestation using causal loop diagrams in an interesting analysis of the feedback mechanisms connecting possible causes and potential effects. These authors mapped out the linkages among variables and drivers of change to shed light on the multiple feedback processes responsible for the dynamic complexity of deforestation. Once this mapping is achieved, however, understanding the effects of the feedback on system dynamics and how it makes variables react to changing conditions (management, policies and perturbations of various origins) remains a challenge. This knowledge is crucial because it can help understand complex responses and unexpected behaviours, the causal nature of which often remains trapped in the intricacy of the interaction pathways.

To better understand the impact of the armed conflict, analyses at a finer resolution that combines social and ecological factors have been called for [18]. Most of the studies in this line of research have focused on statistical models [12,27] that incorporated potential conflict-related drivers as independent variables in linear models that explain variations in the variables of interest, such as, for example, deforestation. Those studies, however, do not reveal the mechanisms through which (statistical) patterns are generated; they allow for the identification of associations between variables rather than causal linkages. In this work, we exploit the structure of the interactions through the method of loop analysis. It allows identifying paths that connect system variables: disentangling such pathways brings to light mechanisms that causally link the system components and explain their response to external events.

## 2. Methods

### (a) The conflict scenario

Land concentration, a heritage of the colonial period, has always been at the heart of Colombia’s internal conflict [28]. In the early 1960s, despite some attempts to attenuate unequal land distribution, access to land was still limited and possible only in peripheral areas [29]. FARC originated in 1964 as a peasant movement in response to this long-lasting rural inequality; more or less contemporarily, another group began its armed insurgency: the National Liberation Army (ELN from the Spanish acronym). Both have been inspired by leftist ideologies, and they had a revolutionary vision against the state, with a political commitment to take power and put into practice a vast, national-scale agrarian reform [30,31].^1^ In response to these left-wing guerrilla groups, ranchers and agribusiness officers financed the constitution of various paramilitary groups that merged into the United Self-Defence of Colombia (AUC from the Spanish acronym), the activity of which was at its maximum level during the 1990s [32,33]. After their demobilization (2005–2007), paramilitarism lasted, and many of their ex-members merged into criminal bands linked to paramilitary ideology [33–35].

Negotiation with FARC has been on the way since 2011 and culminated in the 2016 peace agreement, which was committed not only to rural reform but also to environmental conservation. One main prescription of the agreement was that state lands should be set aside for smallholders to farm. Also, a National Land Fund for victims of forced displacement and land seizure would be created. Other pillars of the agreement comprise redistributing illegally acquired or disused land and the creation of a new registry for recording the property rights over the land across the whole country and areas with special environmental restrictions. Alongside FARC’s concerns over inequality in land tenure, the agreement indicated environmental and biodiversity protection, respect for environmental and human rights and sustainable development as components of the peace process ([36] and refs therein).

The peace with FARC has had, however, unintended consequences. The rebels’ demobilization created the conditions for other insurgents and criminal groups to take control of the areas previously controlled by the FARC. These actors include the National Liberation Army (ELN), which has not signed any peace agreement yet, FARC dissidents (former fighters who have reneged on the peace process and returned to arms) and former right-wing paramilitaries, who ended up merging into criminal groups (Bandas Criminales) [3]. Taking advantage of feeble state authority, those groups have expanded coca growing, cattle ranching, palm oil plantations and other activities at the expense of forested land, sometimes in cooperation with legal actors moved by exploitative ‘business-as-usual’ strategies [37].

The internal armed conflict has heavily affected the socio-ecological context, introducing drivers of change [26] and altering social relationships, with effects on the economy and the environment [6–8]. It has inflated poverty, forced displacements and land grabbing, the effects of which have been amplified by inadequate government policies [38,39]. Also, the armed conflict and the illicit business of coca cultivation interacted, with intertwined effects on the environment [19], as illicit crops have become a funding source for illegal armed groups [14,16–18]. The effects of the conflict on social dynamics are still in force, and the persistence of belligerent factions [4] such as guerilla armed groups, paramilitaries and criminal bands linked to drug trafficking tends to reinforce conflict-related socio-ecological patterns [40].

### (b) Loop analysis

Loop analysis is a qualitative technique that makes use of signed, directed graphs (digraphs) to represent networks of interacting variables [22,41]. The signed digraph depicts interactions between variables by only two types of connections: arrows for positive effects (→) and circle-headed links (⊸) for negative effects of the variables on each other’s rate of change. Loop analysis allows predicting the direction of change in the level (e.g. biomass, number of individuals and spatial extension) of the variables in response to parameter alterations affecting one or more components (i.e. press perturbations; [42,43]). When a press perturbation increases the rate of change of the target variable, this latter is said to undergo a positive input [22]. A negative input occurs when a press perturbation reduces the rate of change of the variable. The effects of an input may percolate to the other components that are connected to the target variable in the network. Their responses are summarized in a table of predictions that accompanies any model (appendices C–E, in the electronic supplementary material). These predictions give only the direction of change for the level of the variables: increase (+), decrease (−) or no change (0). For the sake of simplicity, the table of predictions denotes variations in response to positive parameter inputs (i.e. perturbations that increase the rate of change of target variables). Predictions about negative inputs can be obtained by simply reversing the signs in the table.

In models with a few components and/or a limited number of connections, expected changes for the variables can be tracked through the graph anatomy [44,45]. When variables and connections augment, multiple pathways of interaction emerge, and the probability that they have opposite effects increases (i.e. some paths exert positive effects while others have negative effects) and predictions may remain ambiguous [22]. To overcome this problem, we used a software tool called LevinsAnalysis [24,46], in which a routine performs numerical simulations through the assignment of random values to the coefficients of the community matrix, which is the numerical counterpart of a graph where graphic symbols, arrows and circle-head links are substituted by coefficients +1 and −1, respectively, plus 0 for no interaction. Values are extracted from a uniform distribution in the interval (0, 1, and at each simulation run, they enable calculating path strengths. Hence, even in the presence of multiple positive and negative paths, their cumulative effect is never ambiguous. After the simulations, the algorithm returns the direction of change (increase, +; decrease, –; and no change, 0) for the level of any variable on a percentage base (appendix B). The software tool LevinsAnalysis also executes a stability analysis over the entire set of matrices created in every simulation and returns the number of stable matrices. On this subset of stable matrices, the software program builds the tables of predictions.^2^

Loop analysis has the necessary adaptability and flexibility to be used in socio–ecological analyses. Making predictions in this context is difficult because of the uncertainty associated with new, unknown events, changing dynamics and lack of quantitative data, which frequently occur when studying socio-ecological systems. Loop analysis helps overcome these difficulties as it allows including and discarding variables easily. It permits working with variables and links that are not known or readily measurable, although their effects are crucial. Because information for model construction was collected during a workshop in which stakeholders offered information only in terms of positive or negative effects between the variables, loop analysis seemed the most appropriate tool.

### (c) Correlations from predictions

The table of predictions summarizes the expected responses of the variables following inputs on each and all of them. It may be used as a diagnostic tool in several respects. First, if one knows which variable is going to be affected by a press perturbation (i.e. experiments under controlled conditions; policies or interventions targeted at specific components), the model renders the expected response of all the variables to that input. Such expectations can then be compared with observed variations to assess model reliability. When one does not know where the input(s) enter the system and whether it (they) is (are) positive or negative, model predictions help identify the entry point of the perturbation by examining correlation patterns from predictions [45]. This procedure is detailed in the Appendix D. Unless the system is under control, over a sufficient time interval (or spatial scale [22]), various, not necessarily concomitant, unknown inputs may act on the system; for example, when multiple sources of stress or manifold, uncoordinated interventions, or policies, or simply the effects of the environmental variability occur. Therefore, the trends that variables show can be the net effects of such multiple inputs. In this case, Puccia & Levins [22] suggest looking down any two columns in the table of predictions to deduce expected pairwise patterns of covariation. These predicted covariations can be compared with empirical correlations obtained from the analysis of existing datasets. Such a comparison is possible only if well-defined pairwise covariation patterns emerge from the table of predictions. This occurs only if all the inputs produce coherent variations in any two variables: either they change in opposite directions (negative covariation) or they change in the same direction (positive covariation). If at least one input yields a different covariation between the two selected variables (i.e. they rise and fall together following all the inputs but one, which instead makes one rising and the other falling), then their covariation remains undetermined [22]. To overcome this problem, we simulated multiple inputs on all the variables in the models, randomly changing the combinations of positive and negative inputs over 1000 simulated runs. This procedure renders an overall expectation of change for each variable that we compared to identify pairwise expected covariations. We finally compared predicted covariations with observed correlations obtained by exploiting the proxies that were available for six out of the nine variables that compose the graphs (see appendix D for details about this procedure).

### (d) Model construction

Central to applying loop analysis is the possibility to diagram the structure of the interactions between the system components. First, relevant variables must be identified, and the way they affect each other must be represented in graphical terms according to the loop analysis symbolic language (§2b). Making these interconnections explicit implies translating an idea about the world into a signed digraph. The real complexity of a socio-ecological system cannot be reproduced completely because any modelling effort requires simplifications to achieve an understanding of the system [47]. Using elicitation-based information from a workshop organized on the sidelines of the conference ‘Sustainability, conservation and resilience: strategies for the Colombian post-conflict’, held on March 2018 at the Universidad del Rosario^3^ [13,48], and exploiting the vast literature at our disposal to corroborate that information, we identified a parsimonious set of variables and reconstructed their interactions. We built a series of models that incorporate general, interactive mechanisms that do not take into account features that are specific to different territorial contexts. The models we present here privilege generality to grasp essential dynamics and can be used as core structures for specific applications after refinement. While the appendix A describes in detail the variables and their proxies, in what follows we offer a quick description of the system components.

Forests (F) are essential for food security, livelihoods and natural resource management. They offer opportunities for economic growth and have been at the core of illegal activities. Deforestation, by both legal and illegal actors, has become a pressing problem. All these issues identify forests as a key variable in the Colombian socio-ecological system [9,11,12]. The other ecological component is ecosystem function (EF). It has been included to take into account mechanisms that provide the ecological functions that sustain forests and their biodiversity, and supply ecosystem services [49]. Conflict dynamics are strictly related to land distribution [50,51]. For this reason, we included the following two variables in the models: small property (SP) and large property (LP). The former refers to the small plots farmed for food security and small business by rural people. The latter indicates vast concentrations of land in the hands of a few owners. Both of these variables are represented as dependent on available land (AL), an ancillary variable rather than a real system component. We introduced it to mediate the competition for land between SP and LP. Capital business (CB) refers to activities that are highly capital-intensive, such as oil palm cultivation and banana plantations. The variables that the conflict adds to this framework are violence (V)—a distinctive element of any conflict—and displaced people (DP) because the conflict in Colombia forced a very large number of people to abandon their territories [52]. Illicit business (IB), with its intertwined dynamics with the conflict [20,53], completes the list of the few fundamental variables that form the interactive network of the socio-ecological system as it may have been shaped by the conflict conditions. Variables are summarized in table 1 together with the indicators used as proxies for some of them and their sources of data.

**Table 1.** Core variables used to describe the Colombian conflict-related socio-ecological system.

variable	key	proxy indicator	source of data
forest cover	F	extension of forested areas (ha)	https://ourworldindata.org/grapher/forest-area-km?tab=chart&country=COL
large property	LP	agriculture productive land (ha)	https://data.worldbank.org/indicator/AG.LND.AGRI.K2?view=chart
small property	SP	rural population (ind.)	https://data.worldbank.org/indicator/SP.RUR.TOTL?end=2021&locations/=/CO&start/=/2001&view/=/chart
ecosystem function	EF	biodiversity index (red list index, RLI)	https://stats.oecd.org/Index.aspx?DataSetCode=RED_LIST
capital business	CB	oil palm production (tons)	https://ourworldindata.org/grapher/palm-oil-production?tab=chart&country=COL
illicit business	IB	coca crops (ha)	https://colombiapeace.org/category/infographics/
available land	AL	qualitative	no data
displaced people	DP	qualitative	no data
violence	V	qualitative	no data

When knowledge about interactions is uncertain—because the reports in the literature disagree, information is unavailable or experts suggest different mechanisms of action—plausible, alternative graphs can be reconstructed. After an accurate scrutiny of what the stakeholders suggested in the workshop and the literature, we identified a set of plausible interactions. Appendix E describes these interactions, and table E1 summarizes them graphically. All these interactions designate mechanisms at work in the socio-ecological system under investigation. There is no specific theoretical justification that can help identify a subset of links more reliable than others. Hence, we variously combined them into alternative models that are described in the electronic supplementary material, summarized in table E2 and their full graphical form reported in appendix E. We analysed the entire set of models to find graphs that could better describe the patterns of correlations expressed by the statistical analysis conducted using the proxy indicators. We selected the best configuration based on this analysis and also considered the stability of the simulated matrices.

Model stability was tested within the simulation procedure that computes model predictions: each matrix was analysed for its eigenvalues, which must satisfy the Lyapunov condition (Appendix C). Coherence with observed patterns of correlation was evaluated through a preliminary assessment as described in §3 and detailed in the Appendix D. The models show a gradient of stability (from more to less stable) and also of matching with observed correlations (from higher to lower matching). Model selection was conducted according to these two criteria.

### (e) The FARC and paramilitary/BACRIM scenarios

We recognize that differences exist among the various insurgent groups as for motivation to violence and targets [54,55]. In our models, we considered the role of violence in a very broad sense. We assumed LP as the key generating factor. In fact, it triggered violence directly and indirectly, either because it was perceived as the cause of unequal land distribution (this holds in the case of leftist groups^4^; [56]) or because it deliberately supported right-wing factions for protection against leftist groups, territorial control and land grabbing (the case of right-wing groups [57]). Both of these mechanisms translate into a positive effect from LP to V in the loop analysis language ( LP→V ). Rural people were the main victims of violence. They were deliberately targeted by right-wing groups for land grabbing. Leftist groups, at least at the beginning of their activity, did not target rural people deliberately; nevertheless, their violent actions created risky conditions for rural communities so a negative effect on this part of the population should be considered. In the language of loop analysis, these two actions both translate into a negative link from V to SP ( V−oSP ).

Also, we added V against LP and CB, to simulate the attitude of the left-wing guerrilla groups. In this scenario, the model comprises the following two negative links: from V to LP and CB ( V−oLP;V−oCB ). However, conflict-induced relationships are far more complex than described here [58,59]. We reiterate that the descriptions proposed here may only partially take into account differences in motivation and targets between the many actors of violence. Our models do not dig into the way different ideologies, political and military strategies might have directed the violence in the socio-ecological network [21].

### (f) Data and statistical analyses

The time series for proxies associated with some of the variables in the models are presented in the Appendix B (table B1). We used these time series in a correlation analysis using Pearson’s and Spearman’s statistical tests. We used the Shapiro–Wilk test to assess data normality. The outcomes of the statistical analysis are summarized in table 2 (see §3), and they are given in full in the Appendix B (table B2). The time series span the period 2002–2020. Data are yearly values of the considered quantities. For example, the dataset of forest cover reports yearly values of the extension of the Colombian forests in hectares. Table 1 indicates the official websites from which data were extracted.

## 3. Results

Table 2 summarizes the pairwise correlations obtained in the correlation analysis. The data series and the outcomes of the correlation tests are detailed in the Appendix B.

**Table 2.** Pairwise correlations between the proxies for the model variables listed along the columns. For each proxy the model variable it refers to is indicated in parenthesis. Asterisks identify the level of statistical significance as follows:<0.001 ‘***’; 0.001 ‘**’; 0.01 (*); 0.05 ‘.’; 0.1 ‘ ’. When two symbols/values for the level of significance appear (e.g. (−)*, ns), they refer to the outcome of the Pearson’s test and the Spearman’s test, respectively. Appendix B details the values of the coefficients of correlation for both the tests we executed.

	forest (F)	ecosystem (EF)	rural pop**ulation (SP)**	agriculture (LP)	oil palm (CB)	illicit crop (IB)
**forest**	1	(+)***	(+)***	(−)***	(−)***	(−)*, ns
**ecosystem**		1	(+)***	(−)***	(−)***	(−), ns
**rural population**			1	(−)***	(−)***	(−)*, ns
**agriculture**				1	(+)***	(+)***, ns
**oil palm**					1	(+)***, <0.1
**illict crop**						1

Asterisks identify the level of statistical significance as follows: <0.001 ‘***’; 0.001 ‘**’; 0.01 (*); 0.05 ‘.’; 0.1 ‘ ’.

The normality test and the analysis of the residuals showed that only in some cases were the time series normally distributed, so we performed two correlation tests. All the correlations resulted in significant results applying Pearson’s tests, and only four, all involving data about illicit crops (the proxy for IB), resulted in non-significant outcomes in the Spearman’s test. In a precautionary approach, the results of the non-parametric tests (Spearman) should be taken as the benchmark for the correlations obtained from the model predictions. In the cases of non-significant correlations, however, it is interesting that their sign coincides with those predicted by the models.

Table 3 shows for each model (rows from C1 to C36) the fraction over the 1000 executed runs in which the matching between predicted and observed correlations holds concomitantly for the 15 pairwise correlations. Table 3 also shows the percentage of stable matrices that each model yielded in the simulation.

**Table 3.** Number of times over the 1000 simulation runs that each model yielded the concomitant matching between predicted and observed correlations for all the 15 pairs of variables (match/1000) and the percentages of stable matrices for each model (details are in the appendix C). C28–C36 (in italics) are the counterparts of models C1 to C9 under the FARC scenario.

model	match/1000	stability (%)
C1	278	27.61
C2	360	22.84
C3	439	14.31
C4	224	15.49
C5	280	11.79
C6	455	7.43
C7	580	20.11
C8	482	13.03
C9	427	8.30
C10	219	11.21
C11	346	7.34
C12	170	26.22
C13	204	19.71
C14	227	20.42
C15	290	11.77
C16	448	11.60
C17	475	6.59
C18	370	28.08
C19	483	8.37
C20	440	8.59
C21	173	36.04
C22	54	35.58
C23	81	33.99
C24	38	31.10
C25	93	23.44
C26	27	21.91
C27	266	20.08
C28	26	42
C29	44	39
C30	31	29
C31	12	32
C32	40	29
C33	51	20
C34	43	38
C35	80	29
C36	36	20

**Figure 1.**
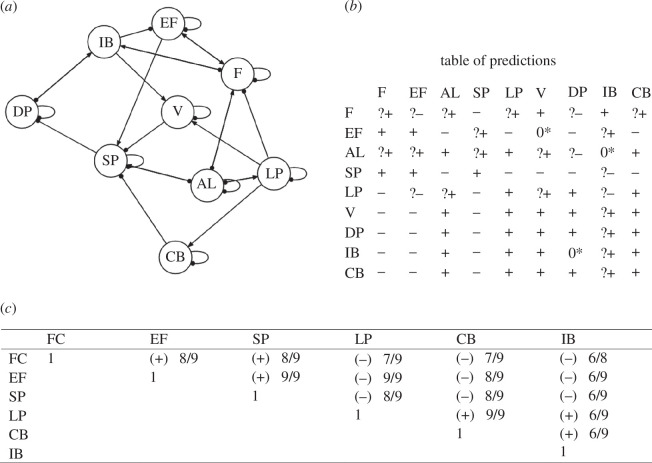
*(a*) The graph of model C7 (appendix E), (*b*) its table of predictions and (*c*) predicted correlations. In the table of predictions, any row lists the response of each and all the column variables when the row variable is affected by (positive) input. Pairwise correlations between any two variables are obtained by looking down their corresponding columns in the table of predictions and comparing for any row (input entering the row variable) the direction of change predicted for the two variables. Numbers quantify the fraction of inputs that yield, for any given pair of variables, covariations that match with the observed correlation, the signs of which are in parentheses. Keys for the variables are as follows: F, forest cover; EF, ecosystem function; SP, small property; LP, large property; CB, capital business; IB, illicit business; V, violence; DP, displaced people; Al, available land. (For an explanation of the question mark symbol, see the appendix C.)

We analysed the entire set of models for correlation matching and stability. We were looking for the best-combined performance of the two attributes, giving priority to the correlation matching, because the agreement with the data is essential for model reliability and the number of stable matrices remains low, even in the case of graphs’ community matrices that are qualitatively stable. The models that compose what we call the FARC scenario (graphs from C28 to C36) show the lowest matching performance, and these models may not adequately describe the system under investigation, although some of them show the highest probability of stability. Models from C1 to C27 describe the paramilitary/BACRIM scenario. They show in general lower stability than the FARC scenario models, but their matching rises noticeably in comparison with the latter. The graph with the highest matching performance is C7: more than 500 (580) runs over 1000 reproduced the observed correlations. Its stability (20%) is lower than that of other graphs, but its combined performance classifies it as the best description that we could obtain for the system. Model C7 is represented in figure 1; below the graph (figure 1*a*) and its table of predictions (figure 1*b*), stands the table of the expected correlations (figure 1*c*) that we obtained from the table of predictions by looking down the columns of the pairs of variables for which we had observed correlations (table 2; Appendix B). Within each pair (columns in the table of predictions), we compared expected variations in response to each and all the inputs to reconstruct their pattern of covariation. The minimum number of inputs for which two variables show the same pattern of covariation and are in agreement with the observed correlations is six out of nine, and this is the case for the pairs involving IB (figure 1*c*, column IB). In two cases (pairs F–LP and F–CB), seven out of nine inputs yielded the same covariation and were in agreement with the observed correlations. For all the other pairs (F–EF, F–SP, EF–SP, EF–LP, EF–CB, SP–LP, SP–CB and LP–CB) eight and nine inputs out of a total of nine rendered the same type of covariation. Because for some couples of variables, the covariation type was not the same for all the nine possible inputs, we performed the simulations as described in §2 and in the appendices C and D. The results of the simulations and the analytical reconstruction of correlation patterns show good coherence. Although only in 580 out of 1000 of the runs the expected correlations matched with the corresponding observed correlations, this result can be considered satisfactory, given the low probability that the match holds for 15 pairs of variables concurrently.

It is interesting to note that the less robust patterns (correlations involving IB) correspond to the cases in which the non-parametric correlation test was non-significant (table 2; Appendix B).

## 4. Discussion

### (a) Model selection

We analysed a series of signed digraphs representing the web of conflict-related social–ecological relationships in Colombia. These structures are based on information gathered in a participatory approach that we validated using the literature, and, in principle, all the models could be reliable. Using observed correlations between proxies for six out of the nine variables in a benchmarking exercise, we searched for better representations of the system among the 36 plausible alternatives. In addition, stability analysis was employed as a further criterion for model assessment. After this scrutiny, model C7 seemed the most reliable description of the system, as it showed the best-combined performance of correlation matching and stability.

Figure 1*c* shows the expected correlations for the pairs of variables for which we computed the correlations from the dataset. We obtained expected correlations using the method suggested by Puccia & Levins [22] and that we described in §3 and in the Appendix D. Correlations emerged clearly in the cases of EF–SP, EF–LP and LP–CB because in each of these pairs, the variables show constant patterns of covariation for each and every input entering the systems. For example, in nine cases over nine possible inputs (i.e. entering anywhere in the system), EF and SP vary in the same direction, so that they covary positively (figure 1*b*; columns EF and SP). This match holds true also for the pairs EF–LP and LP–CB (figure 1*c*). Expected covariations for the other pairs are less defined, but discrepancies are minimal: in the couples F–EF, F–SP, EF–CB, SP–LP and SP–CB, eight of nine inputs yield the same type of covariation. In the cases of F–LP and F–CB, variables’ covariation is the same for seven of nine inputs. Pairs that include IC show less certain covariations because six of nine inputs produce the same covariation between the two variables. When patterns of covariation are not the same for each and every input, Puccia & Levins [22] posited that the overall expected correlation between two variables remains uncertain. To overcome this uncertainty, we simulated concurrent inputs on all the variables, assigning randomly the sign to each input (positive or negative). By simulating different combinations of inputs 1000 times, we obtained a probability of matching the observed correlation. This simulation allowed us to apply for the first time, to our knowledge, the method proposed by Puccia & Levins [22] to assess model reliability using observed correlations as a benchmark. In this way, we could overcome the uncertainty that occurs when different patterns of covariation characterize two variables in their response to the inputs entering the system.

The matching with the observed correlations suggests that model C7 (figure 1) more reliably describes the real system. Its table of predictions, in fact, no matter which variable would be affected by an input or whether multiple inputs would occur (and no matter if they are concomitant or not), highlights that the covariations between the variables are largely always the same.

### (b) Model predictions

Predictions can be used as diagnostic tools for the effects of various inputs that can be associated with policies or interventions targeted at specific variables. Oil palm cultivation has been expanding in the country, favoured by the national policy of subsidized credit, mandatory biodiesel blends and tax exemptions [38,60,61]. These actions enter the model as positive inputs on CB, and variations expected in the level of the model’s variables are listed in the last row of the table of predictions (figure 1*b*). Expectations are for a reduced extension of forested land and associated ecological functions, as well as a contraction of the small farming (figure 1b, row CB: column F, effect (−); column EF, effect (−); column SP, effect (−)). The negative prediction on F is coherent with the effects produced by oil palm development on forests and that are documented in the literature ([38,62], but see [63]) and with the predictions about the future expansion of oil palm cultivars under the business-as-usual scenario [64,65].

The augmented rate of oil palm cultivar establishment is predicted to increase forced displacement, coupled with the loss of traditional farming practices. A positive input on CB would increase DP (figure 1b, row CB; column DP, effect (+)). A causal interpretation of this effect can be obtained by the anatomy of the paths that connect CB and DP (appendix F). Two of these paths make DP increase^5^: CB−∘SP−∘DP and CB−∘SP−∘AL→LP−∘F→IB−∘DP , whereas CB−∘SP−∘AL→F→IB−∘DP would decrease DP. This predicted increase of DP is in agreement with the observation that oil palm development has been increasing internal displacement [61,66,67]. Powerful lobbying and financial capacity that characterize CB, rather than violence, seem to play a major role in increasing internal displacement, as variable V does not enter into the paths responsible for this prediction. Also, large property (LP) contributes to this outcome, possibly in association with illicit business (IB), although the latter also occurs in the path that would decrease DP.

The path responsible for the negative effect on SP of the positive input to CB (see the appendix F) is the link CB−∘SP . As said, this link expresses the negative influence that capital business exterts on small farming, because of its powerful lobbying and financial capacity that easily expropriate land, as well as owing to its competitive advantage for property rights [67]. To counteract this effect, action on the property rights system and trade agreements would be required, a conclusion presented with circumspection. Predictions of a positive input on CB confirm that national policies and trade agreements were beneficial for strongly profitable land uses (e.g. capital-intensive practices, such as oil palm cultivation, rather than labour-intensive ones) at the expense of small-scale producers, ultimately worsening their condition and exacerbating social inequality and environmental damage.

Policies favouring CB are also expected to increase the level of violence (V), the LP, as well as the level of CB itself. Although palm plantation establishment has not always occurred through violence and forced displacement, different authors have documented the connections between oil palm plantations, armed groups and violence [61,66,68]. The model confirms that under the conditions imposed by the armed conflict, V and land tenure concentration (LP) are higher where oil palm production developed [61]. These three variables are, in fact, always positively correlated as one may notice scrolling down their columns in the table of predictions. Also, observed correlations show a significant positive association between CB and LP, but no data were available for assessing the association between CB and LP with V.

Boron *et al*. [38] posited that governments at the national and local level should promote agricultural development via individual small holdings rather than large agribusiness, and that policies should be conceived to improve competitiveness and access to markets of small farming. Re-orienting rural policies in this direction has been envisaged as a way to decrease poverty, increase social equity, safeguard food security and maintain higher levels of biodiversity [69–71]. This type of intervention implies a positive input on SP in the model (figure 1b, row SP). An increase in the level of forest cover and the improvement of its associated ecological functions would be expected following this input (figure 1b, column F, effect (+); column EF, effect (+)). Small farming would also increase (+ in the level of SP), bringing about food security and social cohesion [38]. Also, people displacement (DP) would be reduced ( - in the level of DP). To explain why improving small farming could be beneficial for forest cover requires understanding the connections between these two variables, which are detailed in the Appendix F (paths from SP to F). The effects of these paths can be causally disentangled by considering the predicted changes in the level of each variable making the path, in combination with the effects carried by the links that form the path itself. As many as four paths connect the two variables. The shorter (and stronger in magnitude; see Appendix F) would decrease F ( SP−∘AL→F ) but the other three are all beneficial to it. Of particular interest are the paths SP(+)−∘DP(−)⟶IB(−)−∘F(+) and SP(+)−∘DP(−)⟶IB(−)−∘EF(+)−∘F(+) . The signs in parentheses indicate the expected changes for the variables as they are reported along the row SP of the table of predictions. In the former path, SP has a negative effect on DP because SP facilitates settlement and social cohesion, so DP would be decreasing. This in turn is expected to reduce the working force at disposal of IB, which would be decreasing as well, with a beneficial effect on F as this latter component would be released from the negative pressure of IB. The latter path acts through the improvement of EF (ecosystem functions): reducing IB would bring about less pollution and environmental damage from its environmentally unregulated processes of production. Thus, increasing facilities for small farming may be beneficial to forests because reducing displacement would lower the intensity of IB activity, which exploits forests and inhibits ecosystem functions by polluting the environment.

This intervention would also be advantageous on the side of contrasting illicit business (IB) and violence, as both IB (figure 1*b*; row SP, column IB, effect (−)) and V (figure 1*b*; row SP, column V, effect (−)) are expected to diminish. The shortest and strongest path connecting SP to IB is SP(+)−∘DP(−)→IB(−) , and its sign is negative. The dynamic effect of this path can be reconstructed as well: the beneficial intervention on SP (positive input) would augment its level (figure 1*b*; row SP, column SP, effect (+)). Because SP negatively affects DP, this latter would be decreasing (figure 1*b*; row SP, column DP, effect (−)). Finally, given that DP enhances IB, the expected reduction of DP would have a negative effect on IB, which relies on DP for workforce. Five pathways connect SP to V, and four of them are responsible for the expected reduction of violence following the positive input on SP (appendix F). In the path SP(+)−∘DP(−)→IB(−)→V(−) , the last step describes IB as enhancing violence: because IB is predicted to decrease (see the effect of SP on IB discussed above), its positive effect on violence would also lower, so that V would be diminishing.

The years following the signing of the peace agreement have seen an increase in deforestation in the whole country ([13,72–74], but see [75,27]). Clerici *et al.* [13] found that of 39 protected areas in the country, 79% have undergone higher deforestation rates during post-conflict periods. Also, Negret *et al*. [19] posited that a positive relation between forest cover and the intensity of the armed conflict existed [14], and vast territories formerly under FARC control became accessible to exploitation. The growing warfare ecology literature reports, however, both negative and positive effects of conflict on biodiversity and the natural environment, and ascertaining the overall effect of the conflict on deforestation is controversial [76]. It may have slowed down human penetration into the forests [77], reducing exploitation, but at the same time, it may have caused unsustainable use of forest resources for war efforts [76]. Clerici *et al*. [13] show that both positive and negative effects would depend on the specific complex socio-ecological dynamics linked to the conflict itself. The models analysed here may help envisage possible mechanisms.

Interventions to reduce violence, such as ceasefires, and combatant demobilization enter the model as a negative input on V. Consequences are a beneficial effect for both F and EF, which are expected to increase^6^ (figure 1*b*; row V, column F, inverted sign (-)→(+) ; column EF, inverted sign (-)→(+) ). On the other hand, in the graphs depicting the FARC scenario (models from C28 to C36 in the appendix D), halting the conflict would increase deforestation while inhibiting ecosystem functions. These results would confirm what certain scholars [13,74] posited, but are contrary to what has been documented by others [78]. Basically, the differences between these models stand in the way they incorporate the dynamics of the violence. In the FARC scenario, violence is targeted not only to SP but also to LP and CB. Releasing these two latter components from the pressure of armed groups would increase their exploitative action on forests, with negative effects on ecological functions and biodiversity. However, the models we used to represent the FARC scenario showed a very low agreement with the observed correlations, and as such, we judged them less reliable to represent the structure of the socio-ecological system under investigation. This would suggest that the structure presented in figure 1 most likely reflects the dynamics that characterized the Colombian socio-ecological system, thus confirming the hypothesis that reducing violence would be beneficial for forests and ecological functions, a conclusion presented with circumspection.

IB plays a major role in this conflict-related socio-ecological system. Major environmental problems, such as water and soil contamination owing to the use of agrochemicals and deforestation, were documented together with violence and forced displacement [79]. Assuming an increasing demand for coca by the illegal market (positive press perturbation on IB), the model predicts reduced forest cover and inhibition of ecological functions, while violence and IB would thrive (figure 1*b*; row IB, columns F effect (−), EF effect (−), V effect (+), IB effect (+)). Also, capital business is expected to increase (figure 1*b*; row IB, column CB (+)). The analysis of the paths explains this outcome. There are as many as five paths connecting IB to CB, and all of them are positive (see the appendix F). The action of IB is mediated by the violence, as the path IB(+)→V(+)−SP(−)−oAL(+)→LP(+)→CB(+) shows: an increased production rate of coca would enlarge the related business (IB +), which enhances violence, with negative effect on SP; more land thus becomes available to LP, which favours CB. In the other four paths (appendix F) IB favours CB through deforestation and inhibition of the related ecosystem functions. For example, one of these path is $IB(+)-oF(-)-oAL(+)\to LP(+)\to CB\left(+\right)$ : increased coca production will occur at the expense of forested land, making land available to be exploited by LP, with beneficial effect on CB. Although increasing available land would also enhance SP, the effect on SP is, however, the opposite. Appendix F shows that nine paths connect these two components; only two of them would favour SP through the increasing of available land. The seven paths that would reduce SP also include the strongest ones: IB(+)−oEF(−)→SP(−) and IB(+)→V(+)−SP(−) . In the former, IB negatively affects SP through disrupting the productive base of rural farming. In the latter, violence generated by illicit activities targets SP.

After demobilization and the peace agreement, the behaviour of the armed groups seems to have become more business-oriented and less ideologically motivated, so the model in figure 1 seems appropriate to describe the present status of the Colombian socio-ecological system. The analysis of the paths, associated to model predictions, highlights the mechanisms through which licit and illicit business are inextricably linked one another while conflict conditions persist.

Model outcomes support the hypothesis that, in the context shaped by the conflict, policies promoting capital-intensive economic activities likely would bring about deforestation while weakening ecosystem functions and the associated biodiversity. Also, more unexpectedly, such policies would not improve social cohesion (they would reduce SP) but would enhance conflict dynamics (they would increase V) as well as IB. Our model highlights the potential synergy between the objectives of protecting forests and promoting small farming (positive correlations between EF, F and SP). Enhancing small farming practices (positive input on SP) by specific policies, on the other hand, may not be beneficial for CB, a conclusion presented with circumspection. Small farming-oriented policies would reduce violence instead, as well as people’s displacement and IB. Trade-offs occur when desirable goals cannot be achieved concomitantly: combining increasing profitability and income with reducing deforestation seems impossible through capital-intensive activities. On the other hand, our model indicates a potential synergy between reducing deforestation, increasing social cohesion and decreasing violence and IB.

### (c) Limitations and assumptions of the study

The graphs presented here certainly do not detail all the mechanisms at work in the Colombian socio-ecological system. Any socio-ecological system is challenging to model because its vast array of interactions confounds causes and effects. Using a parsimonious set of variables, we reduced the complexity of the system to a level that allows the unveiling of general mechanisms. We did not consider, for example, government action, urbanization and infrastructure development, although these components are certainly crucial. As for the former, rather than including it in the graphs as a variable, we considered policies and interventions as press perturbations on some components. This allowed avoiding major structural complexity of the graphs. Urbanization and infrastructure development were excluded because a defined pattern of interactions with the other variables did not emerge clearly without considering context-related factors (i.e. urban versus rural dynamics) [48]. Simplification is both legitimate and necessary, provided that we exercise caution, change original assumptions and build new models as needed, and carefully interpret the model’s predictions.

FARC models (C28–C36) show a very low match with observed correlations, indicating they do not capture correctly the main interactions at work under that specific scenario, so that the graphs’ architecture needs to be revised. In particular, the way violence targets the system components will require further, in-depth analysis. Also, a complete dataset (time series for at least eight out of nine variables, including data about violent events and number of displaced people) will be necessary to make the matching analysis more robust. Nevertheless, we think the time series available for six out of nine variables imposes sufficient constraints (the concurrent matching of 15 correlations) to make model selection sufficiently reliable.

The approach we employed responds to the need of ‘dissecting and harness complexity’, as Ostrom [80] posited, but we have done it differently with respect to what the various frameworks used to investigate socio-ecological systems suggest [81]. For example, Ostrom’s framework (socio-ecological system framework (SESF)) is made operational [82] by reconstructing a multi-tier hierarchy of variables relevant for the system. A set of indicators based on primary data pertaining to each variable is constructed, and because the tiers and the associated variables are nested, indicators of lower-tier variables are used to calculate scores for the upper(first)-tier variables. The relationships between the latter are then supposed on the basis of their statistical associations (correlation, regression and principal component analysis) [82].

In the SESF approach, system complexity remains somewhat hidden underneath the statistics, as it does not reveal the mechanisms through which statistical patterns are generated, and variable associations rather than causal linkages can be deduced. By exploiting the interactions network and mapping out the pathways of interactions, the chains of causally ordered events that produce the observed effects may emerge, unveiling interdependencies between variables and not only association.

Despite these differences, however, we see the potential for integration between the two approaches, as shown in a case study of multispecies, multi-fleet coastal small-scale fisheries in the Vizcaino region in Baja California Sur, Mexico [23].

## 5. Conclusions

This article is an attempt to disentangle the complex ties that conflict dynamics may have generated in the Colombian socio-ecological system. Exploiting information obtained through an elicitation approach, we reconstructed a web of socio-ecological interactions involving a parsimonious set of variables. To select the most representative structures, we compared predicted correlations from the models with observed correlations from the time series of proxies for six out of the nine variables we included in the model. In this procedure, we applied, for the first time to our knowledge, the method that derives predicted correlations from the table of predictions of the signed digraphs, combined with a simulation in which sources of variations (inputs) were randomly assigned to each and every variable in a model. Comparing expected with observed correlations offered a realistic criterion for selecting the most reliable model among several (36) plausible structures that came out of the elicitation approach and the literature analysis. Through this procedure, we achieved the objective of shaping a plausible web of socio-ecological interactions (the first specific objective of the work).

We exploited the algorithm of loop analysis to predict the response of the variables to policies or interventions that we simulated as press perturbations (inputs) targeted to specific variables (the second objective of this work). These responses are the outcomes of multiple paths that propagate effects from the entry points of the press perturbations (which act as drivers of change) to the rest of the system. By disentangling the paths and observing the responses of the variables along each path, we have been able to hypothesize the chains of causally ordered events that produced the effects associated with the drivers (e.g. the policies, third objective of this work). In this way, loop analysis offers an opportunity to identify causal linkages wherever pathway multiplicity creates intricate interactive scenarios.

By analysing the response of the variables to inputs, we could shed light on trade-offs and synergies (the fourth specific objective of this work). This search is useful to identify possible win–win solutions that in this specific system seem to be unlikely. In the end, this work, which must be considered a preliminary exploration of the complex conflict-related ties in the Colombian socio-ecological system, clearly suggests that the persisting conflict conditions make licit and illicit activities inextricably linked to one another and that planning without taking this into account may fail to achieve the desired goals.

## Data Availability

Data are provided in the supplementary material [83].

## Footnotes

^1^
The present paragraph summarizes essential elements of the Colombian conflict, which is far more complicated and complex than described here. For detailed, comprehensive reporting about Colombia’s internal conflict, one should refer to these references.

^2^
For the scripts that compose the software tool for the simulations, see [46]

^3^
The participatory workshop that was organized following that event and entitled ‘Future scenarios: tools to create a better future’ saw the participation of experts, mostly from the academic world, in the fields of socio-ecological systems, urbanization, land-use change and deforestation. Through an open discussion, the workshop addressed the issues related to the post-conflict, socio-ecological dynamics, deforestation and land use in the peri-urban environment.

^4^
The main representatives of the left-wing groups are the Fuerzas Armadas Revolucionaria de Colombia (FARC) and the Ejercito de Liberacion Nacional; the right-wing guerrilla groups are the various paramilitary factions that constitute the Autodefensas Unidas de Colombia (AUC).

^5^
The effect that a pathway carries is simply the sign of the product of the links that make the path.

^6^
It must be remembered that the outcomes of negative inputs can be obtained by simply reversing the signs in the table of predictions.
